# Quantitative analysis and visualization of live spotted seabass (*Lateolabrax maculatus*) flesh texture using hyperspectral imaging and machine learning

**DOI:** 10.1016/j.fochx.2026.103773

**Published:** 2026-03-31

**Authors:** Shuai Che, Ang Li, Huan Wang, Changting An, Xiang Fang, Qing Wang, Qiyou Tao, Yun Li, Changlin Liu, Shufang Liu, Zhimeng Zhuang

**Affiliations:** a State Key Laboratory of Mariculture Biobreeding and Sustainable Goods, Yellow Sea Fisheries Research Institute, Chinese Academy of Fishery Sciences, Qingdao 266071, China; b Laboratory for Marine Fisheries Science and Food Production Processes, Qingdao Marine Science and Technology Center, Qingdao 266237, China; c Fujian Minwell Industrial Co., Ltd., Ningde 355200, China; d South China Sea Fisheries Research institute, Chinese Academy of Fishery Science, Guangzhou 510300, China; e Ocean University of China, Qingdao 266003, China

**Keywords:** Hyperspectral imaging, Live spotted seabass, TPA parameters, Machine learning, Texture visualization

## Abstract

Flesh texture is one of the most important quality traits determining consumer satisfaction and perception of fish. Traditional methods for measuring flesh texture characteristics present several limitations. This study developed a rapid, efficient and non-invasive approach based on hyperspectral imaging (HSI) technology for evaluating texture profile analysis (TPA) parameters in live spotted seabass. Three types of surface hyperspectral data (skin with scales, SWS; skin without scales, SOS; and reversed skin without scales, RSOS) were collected from the dorsal region of 300 live fish across 400–1000 nm wavelength range. After applying five spectral pre-processing methods, five machine learning algorithms, including partial least squares regression (PLSR), least square support vector machine regression (LS-SVR), random forest (RF), convolutional neural network (CNN) and back propagation artificial neural network (BP-ANN), were performed to construct optimal prediction models for seven TPA parameters. Gumminess was predicted most effectively, with the RF model achieving a prediction set coefficient of determination (R^2^_P_) of 0.923, a ratio of performance deviation (RPD) of a mean absolute percentage error (MAPE) of 6.378%. The optimal models enabled quantitative prediction of gumminess, chewiness, and hardness (RPD > 3.0 and MAPE<10%). For cohesiveness and springiness, the models performed satisfactory with R^2^_P_ values of 0.859 and 0.854, RPD values of 2.890 and 2.613, and MAPE were 6.858% and 3.002%, respectively. However, prediction accuracy for adhesiveness and resilience was lower, with R^2^_P_ values of 0.552 and 0.341, and RPD values of 1.494 and 1.134, respectively. Visualization maps generated from the optimal models facilitated the direct assessment of TPA parameters distribution in the muscle. This study demonstrated that HSI combined with machine learning offers an effective and non-invasive method for assessing flesh quality in live spotted seabass, thereby accelerating the selection of germplasm with superior textural attributes.

## Highlights

• Hyperspectral imaging was proposed to predict texture characteristics in live spotted seabass.
• RSOS (reversed skin without scales) spectra yielded optimal predictions for hardness, chewiness, gumminess, springiness and cohesiveness, while SOS (skin without scales) data was best for adhesiveness and SWS (skin with scales) data for resilience prediction.
• Hardness, chewiness, and gumminess could be quantitative analysis by optimal models (R^2^_P_ > 0.9, RPD > 3.0, MAPE<10%).
• Models for cohesiveness and springiness are suitable for qualitative analysis (R^2^_P_ ∼ 0.85, RPD ∼ 2.7).
• TPA parameters in dorsal region of spotted seabass were visualized by pseudo-color maps.

## 1 Introduction

Marine fish, rich in high-quality nutrients such as proteins, polyunsaturated fatty acids, vitamins, essential minerals, and trace elements, are increasingly regarded as a healthier alternative to terrestrial meat, helping to meet the global demand for nutritious food amid a growing population (FAO, 2024; Sharma et al., 2025; Tacon, 2023). In recent years, aquaculture operations have increasingly prioritized fish quality in response to the rising consumers' demand for high-quality flesh products (Wang et al., 2023; Jiang et al., 2022). Key flesh quality traits primarily encompass sensory characteristics; nutritional compositions and safety parameters (Matarneh et al., 2021). Notably; flesh texture; encompassing the structural; mechanical; and surface properties of meat products perceived during chewing; is considered the most important sensory factor influencing consumer satisfaction and perception (Wang et al.; 2023; Cheng et al., 2014). It is characterized by series of parameters such as hardness; adhesiveness; resilience; cohesiveness; springiness; chewiness; gumminess; and tenderness (Szczesniak, 2002).

Spotted seabass (*Lateolabrax maculatus*) is widely cultivated along the Chinese coast due to its excellent adaptability to a broad range of temperatures and salinities, delicate taste, and high nutritional value (Huang et al., 2025; Sun et al., 2024). In 2024, its annual production exceeded 257,000 tons, accounting for approximately 12% of China's marine fish aquaculture output (Bureau of Fisheries, Ministry of Agriculture and Rural Affairs of People's Republic of China, National Fisheries Technology Extension Center, China Society of Fisheries, 2025). Diverse cultivation areas and multiple cultivation modes (such as freshwater ponds; seawater ponds and seawater net cages) have resulted in varied flesh texture characteristics (Huang et al., 2025). For spotted seabass, flesh texture diversity not only influences sensory properties but also dictates determines post-harvest processing methods and product value.

Traditional texture assessment methods primarily rely on sensory evaluation and texture analyzer (Ma et al., 2017; Rosenthal & Chen, 2024). Texture profile analysis (TPA) remains a standard method for fish texture characterization (Cheng et al., 2014; Rosenthal & Chen, 2024). Although conventional methods provide benchmark accuracy, they are labor-intensive, time-consuming, require extensive sample preparation, and are operator-dependent (Cao et al., 2023). Moreover; destructive sampling prevents real-time monitoring and can introduces representativeness errors; particularly in heterogeneous matrices (Feng et al., 2025). Therefore, developing rapid, efficient and non-invasive approaches for flesh texture evaluation is imperative.

Over the past decades, the integration of sensor technology and artificial intelligence has established hyperspectral imaging (HSI) as a promising approach for efficient and non-invasive detection combined with machine learning (Che et al., 2024). HSI integrates imaging and spectroscopy to generate three-dimensional (3D) hypercubes containing two-dimensional spatial information and one-dimensional spectral information (Zuo et al., 2024). Unlike Red-Green-Blue systems that capture only three broadband channels in visible range; HSI acquires a continuous spectral data across visible (Vis; 400–700 nm); near-infrared (NIR; 700–1000 nm); and shortwave infrared (SWIR; 1100–2500 nm) wavelengths with high spectral resolution (Bhargava et al., 2024). Therefore; HSI could provide comparatively finer data from the scenes and more informative inputs for subsequent analysis across hundreds of bands (Allentoft-Larsen et al., 2025). The detailed spectral information in hypercubes enables the application of machine learning (ML) or deep learning (DL) methods; including partial least squares regression (PLSR); support vector machine regression (SVR); random forest (RF); convolutional neural networks (CNN) and backpropagation artificial neural network (BP-ANN); to build robust models linking the hyperspectral data to the phenotypic traits (Saha et al.; 2021). Since HSI-acquired signal often contains noise; dark current; and stray light; spectral preprocessing in commonly employed to reduce variability and enhance data homogeneity before modeling (Ravikanth et al., 2017; Zuo et al., 2024).

In recent years, the application of HSI combined with ML for fish quality assessment has gained significant attention, particularly for authenticity (Qin et al., 2020; Xia, Li, et al., 2025), color (Wang et al., 2022; Xia, Xiao, et al., 2025), flavor (Sun et al., 2023); freshness (Moosavi-Nasab et al., 2021; Shao et al., 2023), nutritional parameters (Dixit & Reis, 2023; Zhang et al., 2020), texture (Tang et al., 2024; Wang et al., 2019) and microbial contamination (Khoshnoudi-Nia et al., 2018; Yuan et al., 2025). However, most studies focused on processed fish fillets, and required muscle preparation. The literature on HSI for quality assessment in live fish is limited. Cao et al. (2023) successfully predicted textual parameters in live *Cyprinus carpio* L. using scaled skin HSI (400–1000 nm) and ML; demonstrating excellent performance in gumminess; cohesiveness; adhesiveness; and chewiness. Similarly; HSI accurately predicted PUFA and EPA + DHA content in live common carp (Cao et al., 2024). Nevertheless, to our knowledge, no such study has been conducted on live maricultured fish.

This study aims to establish a rapid, efficient, and nondestructive method to replace traditional techniques for evaluating flesh texture in live maricultured fish. We investigated the potential of HSI (400–1000 nm) combined with machine learning for detecting the muscle TPA parameters in live spotted seabass. The specific objectives were: (I) synchronous acquisition of three hyperspectral data and seven muscle TPA parameters; (II) application of five spectral preprocessing methods to reduce data variability and enhance spectral features; (III) determination of optimal relationship between HSI data and TPA parameters using five ML methods; and (IV) the visualization of TPA parameters distribution in muscle based on the optimal prediction models.

## 2 Materials and methods

### 2.1 Ethics statement and sample preparation

**Fig. 1:**
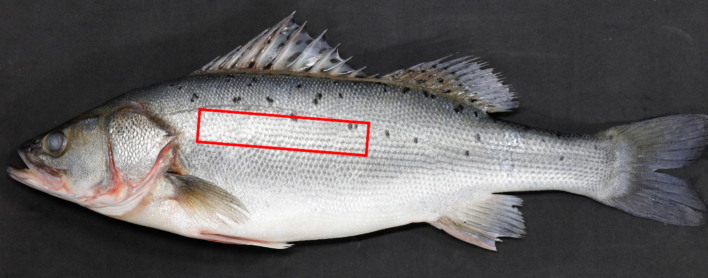
The target dorsal region (within the area indicated by the red rectangle, 8 cm × 2 cm × 1 cm) of spotted seabass used in this study. (For interpretation of the references to color in this figure legend, the reader is referred to the web version of this article.)

All procedures were conducted in accordance with the Regulations on the Administration of Laboratory Animals (Third Edition, 2017) of the People's Republic of China and were approved by the Institutional Animal Care and Use Committee of Yellow Sea Fisheries Research Institute, Chinese Academy of Fishery Sciences (Approval code: YSFRI-2024042 on 8 March 2024). Prior to sampling, all spotted seabass were reared in commercial aquaculture farms located in the three major aquaculture regions representing the primary production areas for this species: Rizhao (Shandong Province, avg. water temp: 16.43 °C; salinity: 31‰), Fuding (Fujian Province, avg. water temp: 20.92 °C; salinity: 18‰) and Zhuhai (Guangdong Province, avg. water temp: 25.48 °C; salinity: 1.5‰) (Table 1). Fish were fed a combination of commercial formulated feed and ice-fresh trash fish. According to previously reported methods (Cao et al., 2023; Ma et al., 2017; Rajput et al., 2023; Tang et al., 2024), a total of 300 commercially sized spotted seabass young fish (weighting approximately 700–1000 g) were randomly netted from culture cages without selection based on size or condition following their routine harvesting procedure. Sample sizes were allocated according to regional production volumes, with the largest number from Zhuhai (*n* = 120), followed by Fuding (*n* = 105), and Rizhao (*n* = 75). No formal a priori power calculation was performed because the study objective was predictive model development rather than hypothesis testing. All individuals met the following inclusion criteria: intact scales; absence of visible wounds, hemorrhages, or skin lesions on the body surface; no apparent parasitic infection; no spinal curvature or other morphological abnormalities; and normal swimming activity. Sex was not determined because external sexual dimorphism is absent in this species at market size. Fishes were anesthetized using 100 mg/L MS-222 (tricaine methanesulfonate, Sigma Aldrich Chemie GmbH, Steinheim, Germany) for 15 min prior to sampling to minimize suffering. Hyperspectral data and muscle samples were collected from the left dorsal region of each specimen, with the sampling area delineated by the red rectangle (8 cm × 2 cm × 1 cm) as shown in Fig. 1. Each individual fish was considered as an independent experimental unit. Hyperspectral imaging and TPA parameters were acquired on the same fish, and each fish contributed one complete data record to the dataset. After the collection of hyperspectral data and muscle samples, the fish were euthanized using 300 mg/L MS-222.

**Table 1:** The samples information for this study.

Sampling area	Quantity	Weight (g)	Longitude and latitude
Rizhao	75	748.58 ± 140.93	35°29′ N, 119°36′ E
Fuding	105	840.69 ± 145.21	27°12′ N, 120°19′ E
Zhuhai	120	841.74 ± 97.33	22°10′ N, 113°21′ E
In total	300	819.31 ± 132.49

### 2.2 Hyperspectral image acquisition

**Fig. 2:**
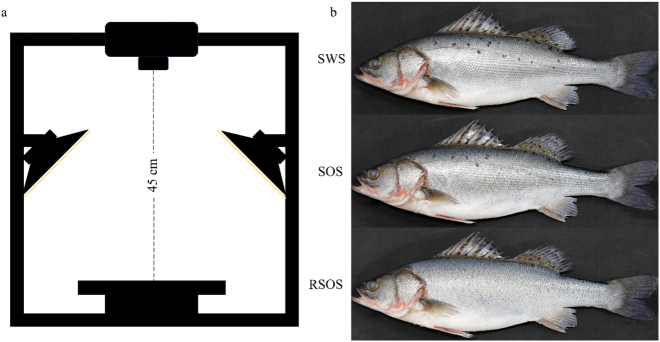
Schematic diagram of the HSI system (a) and three types of hyperspectral data from the spotted seabass (b).

Hyperspectral images were acquired using an HSI system comprising a Specim IQ camera (Specim Ltd., Oulu, Finland), two 375-W halogen lamps (ARRILITE 750 Plus, ARRI, Germany), and a black background substrate (Fig. 2a). All acquisitions were carried out in a dark room to minimize the interference from ambient light. The hyperspectral camera captured 512 × 512-pixel images across 204 spectral bands within 397.32–1003.58 nm at a 7 nm bandwidth. Halogen lamps illuminated the viewing area at 45° angles. Prior to imaging, live specimens were stabilized on the substrate with left dorsal side perpendicular to the optical axis; and a horizontal white reference panel (99% light reflection) was placed beside the sample for radiometric calibration. Three spectral data types were acquired from the wiped dorsal surface: skin with scales (SWS, the skin with scales intact), skin without scales (SOS, the preparation procedure of SOS was as follows: The scales on left side were carefully removed using an abrasive tool. Following scale removal, the skin surface was gently smoothed by wiping from the head towards the tail direction to ensure the removal of any residual mucus or debris.) and the reversed skin without scales (RSOS, the preparation procedure of RSOS was as follows: Following scale removal, the skin surface was gently smoothed by wiping from the tail towards the head direction to ensure the removal of any residual mucus or debris, thereby exposing a clean and consistent epidermal layer.) (Fig. 2b). The lens-to-sample distance was fixed at 45 cm, with an exposure time of 5 ms. Dark and white reference were recorded automatically and simultaneously with the sample, respectively. Image correction was performed automatically using the built-in software. The image data were processed and stored in the default recording mode. The order of hyperspectral imaging was randomized across the same aquaculture region to avoid potential confounding effects related to imaging time. No hyperspectral images were discarded during data acquisition and a total of 900 hyperspectral images were acquired for further analysis. Model development and spectral preprocessing were conducted using anonymized datasets without access to sampling region identifiers.

skin with scales (SWS), skin without scales (SOS) and reversed skin without scales (RSOS).

### 2.3 Image segmentation and spectral data extraction

The average reflectance of the region of interest (ROI, Fig. 1) for each sample was extracted using the Environment for Visualizing Images (ENVI) 5.3 software (Exiles Visual Information Solutions, USA) after converting raw spectral radiance images to reflectance images. The average spectrum of each ROI was calculated from all pixels, excluding shadows and highlights. Given the low signal-to-noise ratio at spectral extremes, 200 bands within 400.32–997.40 nm range were selected for subsequent analysis.

### 2.4 Spectral preprocessing

The preprocessing algorithm could eliminate the impact of light source variations and instrument noise, and highlight the desired spectral characteristics. In this study, 5 preprocessing methods, including Savitzky-Golay (S-G, polynomial order: 2; points of window: 5) smoothing method, S-G + standard normal variate (S-G + SNV), S­G + first derivative (S-G + 1st), S­G + second derivative (S-G + 2nd), and detrend method, were employed before modeling using the Unscrambler X 10.4 software (CAMO, Norway). Spectral pre-processing was performed on all samples uniformly, without consideration of geographical origin.

### 2.5 Texture profile analysis

Muscle samples corresponding dorsal regions (Fig. 1) were harvested and segmented into 2 cm × 2 cm × 1 cm cubes for texture profile analysis. Three cubes per sample were analyzed, and the average value represented the TPA parameters of individual sample. Seven texture parameters, including hardness (g), adhesiveness (g∙s), chewiness (g), gumminess (g), springiness, cohesiveness and resilience, were measured using a texture analyzer (TA. PORTABLE, Baoman, Suzhou, China) with a 50 mm cylindrical probe (TA/50). The probe descended at 2 mm/s, triggered at 5 g force, compressed at 1 mm/s speed to 60% of initial height. Following this, the probe withdrew at 2 mm/s, documenting the sample's recovery dynamics. The compression was repeated twice with an interval of 3 s to generate a force-time curve. TPA parameters were calculated using Bourne's technique. Texture profile analysis was performed in the same order as hyperspectral image acquisition to maintain sample traceability. No TPA parameters were discarded during data acquisition. PCA and correlation analysis were performed using Origin 2024 software (OriginLab, USA) to examine differences among sampling regions.

### 2.6 Multivariate modeling

Before model development, all samples from the three geographically distinct regions (Zhuhai, Fuding and Rizhao) were pooled. The 300 samples were randomly divided into a calibration set and a prediction set in a 9:1 ratio (n_calibratio*n*_ = 270, n_predictio*n*_ = 30) using stratified random sampling method according to Greener et al. (2022). This approach ensured that both the calibration and prediction sets contained representative samples from all geographical origins, thereby preventing the model from being developed or validated on a geographically biased subset. Furthermore, it ensured consistency in the range, mean, and standard deviation of the TPA parameters between the two sets, supporting the robustness and comparability of subsequent modeling results. To investigate the relationship between spectral data and texture parameters of spotted seabass, five machine learning methods were employed, i.e., partial least-square regression (PLSR), least square support vector machine regression (LS-SVR), random forest (RF), convolutional neural network (CNN) and back propagation artificial neural network (BP-ANN). Traditional inferential assumptions are not required for model training but model performance was evaluated using a 10-fold cross-validation scheme to avert model oversimplification and overfitting. All cross-validation folds were constructed at the level of individual fish to ensure independence.

PLSR is one of the most commonly used methods for hyperspectral image data analysis (Sun et al., 2017). It is used to optimize the covariance between the label and linear combinations of features by simultaneously decomposing the multivariate input data. According to our previous work (Che et al., 2023), fifty latent variables were considered for the PLSR model establishment, and the optimal number of latent variables (n_LV_) was ascertained by minimizing the root mean square error of cross validation (RMSECV), which inherently guards against overfitting.

LS-SVR is a nonlinear machine learning method that efficiently performs nonlinear regression using radial basis function (RBF) kernel (Nirere et al., 2022). The regularization parameter *C* and the RBF kernel function parameter g were used to reduce the complexity of the model. The performance of the LS-SVR model depends on appropriate tuning of its two key parameters with the minimum prediction error (Li et al., 2023).

RF is a kind of supervised machine learning method that uses a bootstrap statistical resampling technique to create a collection of decision trees (Saha et al., 2021). In RF, numerous decision trees are applied to different subsets of a dataset, leading to higher precision prediction (Saha et al., 2021). Compared with the instability and substantial variability that may arise when using a single decision tree model, random forests composed of multiple trees overcome these shortcomings, and the number of labels classified by all decision trees directly determines the final prediction of a forest (Ghadernejad & Esmaeili, 2024). The number of tree and the minimum number of samples per leaf nodes were set to 100 and 2, respectively.

CNN is one of the most representative network framework in deep learning which has high robustness and strong generalization ability (Yang et al., 2019). It is a feedforward neural network with convolution computation and deep structure for image analyzing (Lu et al., 2024). The basic architecture of the framework is mainly composed of input layers; convolution layers; pooling layers; fully connected layers and output layers (Yang et al., 2021). In addition; feature fusion is typically performed in deep learning before the fully connected layer; and the most common methods are: (1) point-wise addition and (2) concatenation (Zhao et al., 2021). In this study, point-wise addition was selected for feature fusion. The CNN architecture consisted of two convolutional branches, each comprising a convolutional layer followed by batch normalization and ReLU activation, forming a multi-scale feature extractor for progressive feature extraction. The extracted features were then flattened and passed through a three-stage fully connected regressor to perform the final regression prediction.

BP-ANN is developed to imitate the functioning of human brain and used to train multilayer feedforward neural networks with an error-reversal propagation algorithm (Saha et al., 2021). It consists of an input layer, one or more hidden layers, and an output layer, each containing several nodes. The performance of the BP-ANN model is influenced by several parameters, including the number of hidden layer nodes, learning rate factor, momentum factor and initial weights (Saha & Manickavasagan, 2021). The optimal BP-ANN architecture consisted of two hidden layers with 64 and 32 nodes, respectively. The ReLU function was employed as the activation function. The model was trained using the Adam optimizer with an adaptive learning rate, initialized at 0.001. The maximum number of training iterations was set to 2000.

### 2.7 Model performance evaluation

The performance of the model was evaluated by calibration and prediction coefficient of determination (R^2^_C_, R^2^_P_), the root mean square error of the calibration and prediction set (RMSE_C_, RMSE_P_), ratio of performance to deviation (RPD) and mean absolute percent error (MAPE). Higher R^2^_C_, R^2^_P_ and RPD values, along with lower values of RMSE_C_, RMSE_P_ and MAPE, correspond to better predictive ability and accuracy of the model, showcasing the robustness in estimating the TPA parameters effectively. The R^2^, RMSE, RPD and MAPE values are calculated as shown in following equations.

R^2^ = 1-$\;\frac{\sum {\left(y_{i}-{\hat{y}}_{i}\right)}^{2}}{\sum {\left(y_{i}-{\bar{y}}_{i}\right)}^{2.}}$

RMSE = $\sqrt{\frac{1}{N}\times \sum {\left(y_{i}-{\hat{y}}_{i}\right)}^{2}}$

RPD = SD/RMSE_P_

MAPE = $\frac{\frac{1}{N}\sum \left|y_{i}-{\hat{y}}_{i}\right|}{{\bar{y}}_{i}}\times 100\%$

where N is the number of specimens in the calibration set (270) or prediction set (30); $y_{i}$ and ${\hat{y}}_{i}$ are the lab-measured and model-predicted values, respectively; and SD and ${\bar{y}}_{i}$ are the standard deviation and mean of the lab-measured values.

Python3.9 (with the NumPy, Pandas, PyTorch, and Scikit-learn libraries) was used for data analysis including modeling and model evaluation.

### 2.8 Visualization of the TPA parameters in Spotted seabass

The optimal predictive models for the seven TPA parameters were applied at the individual pixel-level characterization. Spatial distribution maps were generated using Python 3.9 (with the Matplotlib libraries) in this study to visualize TPA values heterogeneity across the dorsal muscle.

## 3 Results

### 3.1 Spectral characteristics of SWS, SOS and RSOS

**Fig. 3:**
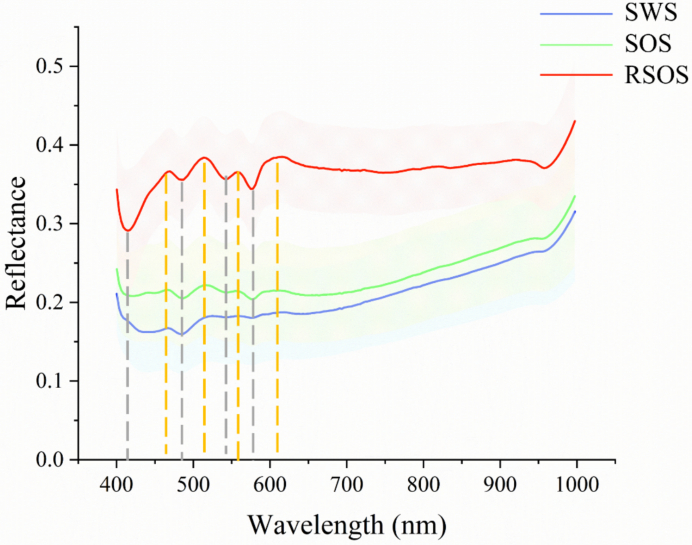
The spectral characteristics of skin with scales (SWS, blue), skin without scales (SOS, green) and reversed skin without scales (RSOS, red) from the dorsal region of spotted seabass (n = 300). The solid lines represent mean reflectance, and the standard deviation range is given bounding with corresponding colors. The yellow dotted vertical lines represent the reflectance peaks and the grey dotted vertical lines represent the absorption peaks in spectra. (For interpretation of the references to color in this figure legend, the reader is referred to the web version of this article.)

SWS and SOS exhibited similar spectral characteristics, distinct from RSOS (Fig. 3, *n* = 300). RSOS reflectance was higher across all wavelength than those of SOS and SWS. Typical absorption peaks in the visible region for all data types occurred at 410, 490, 540 and 580 nm (grey vertical line, Fig. 3), while reflectance peaks were approximate at 480, 520, 560 and 610 nm (yellow vertical line, Fig. 3). An absorption peak at 970 nm was observed in the NIR region for all three data types.

**Fig. 4:**
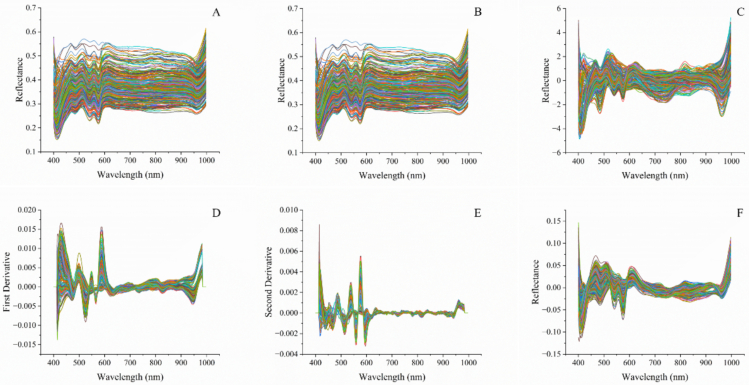
Reflectance spectra of reversed skin without scales (RSOS) samples in Vis-NIR ranges (*n* = 300): (A) raw average, (B) S-G smoothing, (C) S-G + SNV preprocessing, (D)S-G + 1st preprocessing, (E)S-G + 2nd preprocessing, and (F) detrending.

The mean reflectance spectra from 900 ROIs were used for model development. Fig. 4A shows the 300 raw average RSOS spectra. Consistent spectral patterns were observed across specimens from different production region, though overlaps and fluctuations in the original spectra adversely affected the model performance. Preprocessing combination methods, i.e., S­G smoothing (Fig. 4B), S­G + SNV (Fig. 4C), S­G + 1st (Fig. 4D), S­G + 2nd (Fig. 4E), and detrend method (Fig. 4F) were performed to minimize interference. Preprocessing results for SWS and SOS were shown in Supplementary Fig. S1 and Fig. S2.

### 3.2 Diversity of TPA parameters in dorsal muscles

**Fig. 5:**
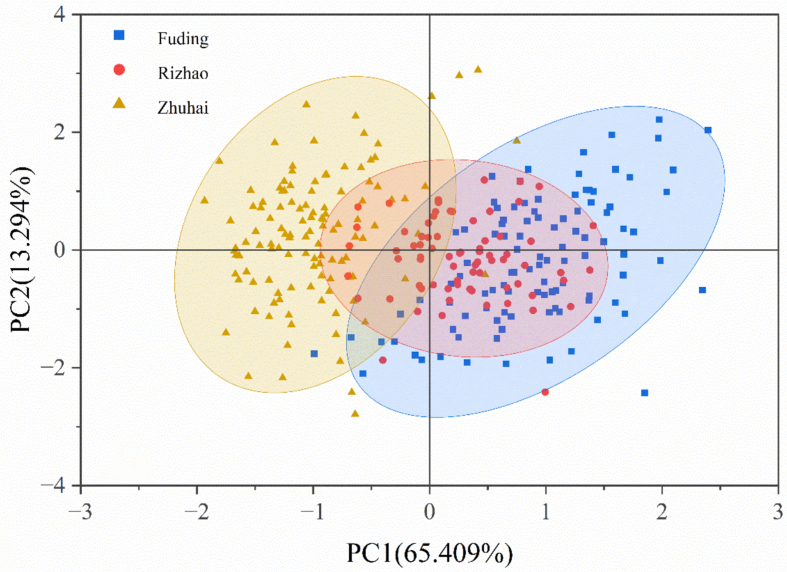
PCA clustering spotted seabass from three production regions based on dorsal muscles TPA parameters (n = 300).

Seven TPA parameters were analyzed for 300 dorsal muscles samples from three production regions (Supplementary Table S1). PCA revealed that the first two principal components explained 65.409% and 13.294% of total variance, respectively (Fig. 5). Samples from Fuding and Zhuhai formed distinct clusters, indicating substantial TPA differences, while Rizhao samples displayed intermediate clustering. Hardness, chewiness, gumminess and springiness were significantly higher in Fuding samples and lowest in Zhuhai samples (Supplementary Fig. S3). Adhesiveness and cohesiveness were significantly lower in Zhuhai samples, with no significant difference between Rizhao and Fuding samples. Resilience showed no significant regional difference (Supplementary Fig. S3). These data revealed the different texture characteristics of the muscle from three production regions.

**Fig. 6:**
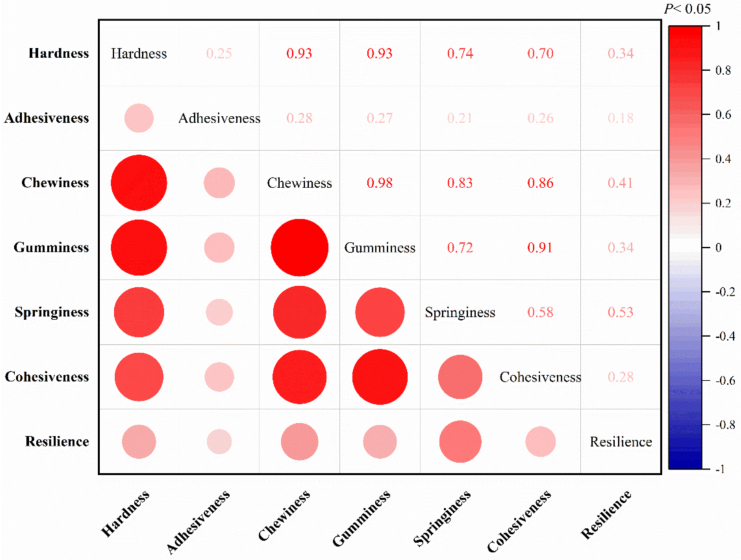
Pearson correlation analysis among TPA parameters in dorsal muscle of spotted seabass (n = 300).

Pearson correlation analysis revealed relationships among all seven TPA parameters (*P* < 0.05, Fig. 6). The highest correlation was between chewiness and gumminess (0.98), followed by hardness and chewiness (0.93), hardness and gumminess (0.93), and gumminess and cohesiveness (0.91). Additionally, strong positively correlations were also observed between chewiness and cohesiveness, chewiness and springiness, hardness and springiness, gumminess and springiness, as well as hardness and cohesiveness, with all correlation coefficients exceeding 0.7. In contrast, resilience and adhesiveness showed relatively low correlations with other parameters.

The descriptive statistics of calibration and prediction sets for seven TPA parameters were shown in Table 2. Chewiness exhibited the greatest variation, ranging from 630.482 to 3945.769 g, with calibration and prediction set coefficients of variation (CV) of 38.936% and 38.600%, respectively. Hardness, adhesiveness, gumminess and cohesiveness all showed substantial variation (CV > 15%), indicating significant heterogeneity among the individuals. In contrast, springiness and resilience demonstrated lower variability (CV < 15%), suggesting minimal difference in these TPA parameters across the specimens. The means and standard deviations were similar between the calibration and prediction sets for all parameters, with no significant distribution difference (*P* > 0.05). Consequently, the prediction sets were applied to evaluate the accuracy of the prediction model generated from the calibration sets for these seven parameters.

**Table 2:** Descriptive statistics of TPA parameters for 300 spotted seabass dorsal muscle samples, 270 for calibration and 30 for prediction.

Constituent	Samples	Range	Mean	Standard deviation	CV (%)
Hardness (g)	Calibration	4049.888–8281.321	6018.668	1017.883	16.912
	Prediction	4611.813–7642.522	6053.434	985.221	16.275
Adhesiveness (g∙s)	Calibration	−8.613–-47.117	−22.447	6.831	30.432
	Prediction	−13.380–-38.517	−21.741	5.587	25.698
Chewiness (g)	Calibration	630.482–3945.769	1912.484	744.639	38.936
	Prediction	881.620–3460.844	1937.823	747.992	38.600
Gumminess (g)	Calibration	1168.179–5241.728	2919.105	927.263	31.765
	Prediction	1633.663–4565.847	2949.773	908.088	30.785
Springiness	Calibration	0.492–0.803	0.639	0.063	9.859
	Prediction	0.519–0.757	0.639	0.064	10.016
Cohesiveness	Calibration	0.265–0.643	0.474	0.088	18.565
	Prediction	0.318–0.643	0.478	0.089	18.619
Resilience	Calibration	0.162–0.338	0.234	0.032	13.675
	Prediction	0.197–0.333	0.245	0.034	13.877

### 3.3 Modeling analysis of TPA parameters based on full spectra

To develop a universal model predicting texture characteristics across major production areas, prediction models were constructed using three types of hyperspectral data (SWS, SOS and RSOS), five spectral preprocessing methods (S­G smoothing, S­G + SNV, S­G + 1st, S­G + 2nd, and detrend method) and five machine learning algorithms (PLSR, LS-SVR, RF, CNN and BPANN) with consistent calibration and prediction datasets. Different combinations yielded various prediction performance (Supplementary Table S2). RSOS data yielded optimal predictions for hardness, chewiness, gumminess, springiness and cohesiveness, meanwhile, SOS was best for adhesiveness and SWS for resilience. RF and CNN were the most effective ML algorithms to predict the TPA parameters (Table. 3). RF achieved best calibration results for adhesiveness (R^2^_C_ = 0.878), chewiness (R^2^_C_ = 0.962), gumminess (R^2^_C_ = 0.968), springiness (R^2^_C_ = 0.923) and cohesiveness (R^2^_C_ = 0.962). CNN was optimal for hardness (R^2^_C_ = 0.971) and resilience (R^2^_C_ = 0.756) prediction, respectively.

**Table 3:** Performance of optimal models to predict TPA parameters using different hyperspectral data types, preprocessing methods, and machine learning algorithms. (optimal models are in bold typeface).

TPA parameter	Hyperspectral data	Optimal preprocessing method	Optimal ML method	Calibration set (n = 270)		Prediction set (n = 30)			
				R^2^_C_	RMSE_C_	R^2^_P_	RMSE_P_	RPD	MAPE
Hardness (g)	SWS	Detrend	RF	0.951	226.349	0.856	451.557	2.631	5.604%
	SOS	S-G + 1st	CNN	0.982	136.323	0.843	463.761	2.526	6.193%
	**RSOS**	**S-G + 1st**	**CNN**	**0.971**	**174.746**	**0.908**	**355.457**	**3.066**	**4.904%**
Adhesiveness (g∙s)	SWS	S-G + 2nd	LS-SVR	0.383	5.074	0.414	4.438	1.306	13.742%
	**SOS**	**Detrend**	**RF**	**0.878**	**2.340**	**0.552**	**4.196**	**1.494**	**12.651%**
	RSOS	S-G + SNV	RF	0.877	2.391	0.459	4.625	1.360	13.948%
Chewiness (g)	SWS	Detrend	BP-ANN	0.777	352.873	0.908	230.421	3.295	11.363%
	SOS	S-G + 2nd	RF	0.958	151.843	0.833	316.227	2.450	13.288%
	**RSOS**	**S-G + 2nd**	**RF**	**0.962**	**144.396**	**0.922**	**212.127**	**3.579**	**9.848%**
Gumminess (g)	SWS	Detrend	RF	0.961	184.121	0.895	312.996	3.080	10.861%
	SOS	S-G + SNV	RF	0.960	183.555	0.861	369.997	2.681	13.204%
	**RSOS**	**S-G + 1st**	**RF**	**0.968**	**165.609**	**0.923**	**235.969**	**3.612**	**6.378%**
Springiness	SWS	Detrend	RF	0.916	0.018	0.833	0.024	2.447	3.014%
	SOS	S-G + 1st	RF	0.907	0.019	0.697	0.034	1.818	4.474%
	**RSOS**	**S-G + 2nd**	**RF**	**0.923**	**0.017**	**0.854**	**0.022**	**2.613**	**3.002%**
Cohesiveness	SWS	S-G + SNV	RF	0.952	0.019	0.781	0.042	2.135	7.963%
	SOS	Detrend	BP-ANN	0.939	0.021	0.847	0.039	2.560	7.002%
	**RSOS**	**S-G + 2nd**	**RF**	**0.962**	**0.017**	**0.859**	**0.037**	**2.890**	**6.858%**
Resilience	**SWS**	**S-G + SNV**	**CNN**	**0.756**	**0.015**	**0.341**	**0.030**	**1.134**	**8.952%**
	SOS	S-G + 2nd	LS-SVR	0.292	0.027	0.122	0.031	1.067	9.064%
	RSOS	Detrend	LS-SVR	0.299	0.026	0.021	0.032	1.048	9.176%

The gumminess was best predicted by the RF model with RSOS spectrum and S-G + 1st preprocessing (R^2^_P_ = 0.923, RPD = 3.612, MAPE = 6.378%). The chewiness was best predicted by RF model with RSOS and S-G + 2nd processing, and the hardness by CNN with RSOS and S-G + 1st. Gumminess, chewiness, and hardness could be quantitatively predicted, as indicated by RPD > 3.0 and MAPE<10%. Cohesiveness and springiness models performed satisfactorily with the R^2^_P_ above 0.8, RPD more than 2.5 and MAPE less than 10%. However, for adhesiveness and resilience prediction, the R^2^_P_ values were low at 0.552 and 0.341 with RPD of 1.494 and 1.134 for the prediction datasets, respectively, which indicates poor prediction insufficient for subsequent analysis and needing further improvement (Table 3).

**Fig. 7:**
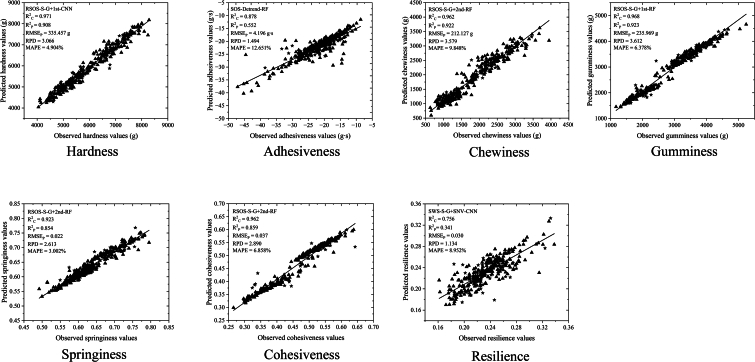
Scatter plots of observed values vs. predicted values of seven TPA parameters using optimal models. Training sets (n = 270) are denoted by ▲ and test sets (n = 30) by ★.

The dispersions between the observed and predicted values of seven TPA parameters using the optimal integrated models (bold in Tables 2) are shown in Fig. 7. RF was the best methods for predicting chewiness, springiness, and cohesiveness prediction using RSOS spectrum with S-G + 2nd preprocessing. Adhesiveness and gumminess were best predicted by the RF with SOS-Detrend and RSOS-S-G + 1st, respectively. Hardness and resilience were best predicted by CNN with RSOS-S-G + 1st and SWS-S-G + SNV, respectively.

### 3.4 Visualization of TPA parameters in dorsal region

**Fig. 8:**
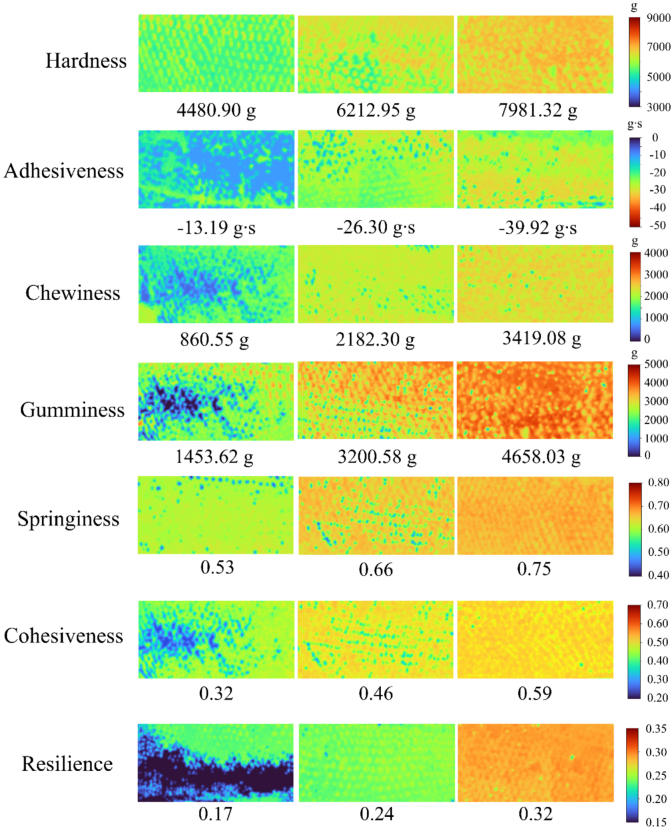
Visualization of the seven TPA parameters in spotted seabass using the optimal prediction models. The left, middle, and right columns correspond to the low, medium, and high value ranges of each TPA parameter, respectively.

The reliable prediction models enabled visualization of TPA parameter variation in the dorsal region (Fig. 8). Samples representing low, median, and high quartile values were selected. Parameter values are encoded in a continuous blue-to-red pseudo-colors gradient, where spectral intensity correlates with magnitude (blue: minimum, red: maximum). Distinct color segregation confirmed significant differentiation among TPA levels. Pixel-level maps revealed non-uniform spatial distribution patterns, with ROIs containing a mixture of high and low values. This visualization allows TPA value estimation at any muscle point, surpassing traditional average-value methods and facilitating selection based on texture attributes.

## 4 Discussion

### 4.1 Flesh texture characteristics of the spotted seabass from different regions

Phenotypic datasets with sufficient dynamic range and precision are crucial for establishing developing universal spectral calibration models (Che et al., 2023). Environmental factors; particularly temperature and salinity; significantly influence fish texture quality by modulating myofiber growth; collagen turnover; and chemical composition (Qu et al., 2022; Wang et al., 2024; Zhang et al., 2021). Spotted seabass, a euryhaline (salinity tolerance: 0–45‰) and eurythermal (temperature tolerance: 0.93–37.10 °C) fish species, is farmed across diverse environment, leading to considerable texture variation among population (Tian et al., 2020; Zhang et al., 2017). Specimens from Guangdong, Fujian, and Shandong provinces, representing ∼87% of national production, showed significant textural differences. Chewiness, hardness, adhesiveness, gumminess and cohesiveness exhibited high variations with CV more than 15%. Specimens collected from Fuding, Fujian province (avg. water temp: 20.92 °C; salinity: 18‰) exhibited superior texture properties compared to Rizhao, Shandong province (16.43 °C; 31‰), which surpassed Zhuhai, Guangdong province (25.48 °C; 1.5‰) samples (Supplementary Table S1, Supplementary Fig. S3). While seawater-cultured spotted seabass typically commands higher market prices (Xu et al., 2010); our results demonstrated that specimens cultured in brackish water exhibit superior sensory properties in muscle tissue. The optimal temperature for the growth of spotted seabass is 23–27 °C (Cheng et al., 2021); comparable to Zhuhai; and the optimal salinity is 16–17‰ (Zhang et al., 2017); corresponding to Fuding. This suggests that the optimal growth temperature does not necessarily yield superior flesh quality; instead; optimal salinity appears more critical for texture enhancement; consistent with findings in Atlantic salmon and other freshwater-farmed fishes (Du et al., 2022; Imsland et al., 2021, Imsland et al., 2019; Qu et al., 2022).

### 4.2 HSI for efficient TPA parameters evaluation

Monitoring textural dynamics is essential for harvesting high-quality fish, but high-throughput methods remain underdeveloped (Fu & Yuna, 2022). The traditional sensory evaluation method for flesh texture depends to large extent on subjective assessments of the expert panel; and TPA requires specialized instrument and skilled operators; with limitations in efficiency and scalability; being time-consuming; destructive; and inefficient for large-scale samples measurement (Cao et al., 2023; Cheng et al., 2014; Tang et al., 2024)). Hyperspectral imaging provides a promising and non-invasive alternative for muscle quality assessment (Feng et al., 2025; Zuo et al., 2024).

Previous HSI studies on flesh texture focused on muscle samples, requiring skin removal (Ma et al., 2017; Tang et al., 2024; Zhou et al., 2020). Recent studies demonstrated the feasibility of skin hyperspectral information for predicting fish muscle quality (Cao et al., 2023; 2024). HSI enables concurrent modeling of multiple properties from a single-image; enhancing throughput and reducing the cost of the measurement (Che et al., 2023). This study confirms the potential of surface HSI combined with ML methods for predicting the muscle TPA parameters in live spotted seabass. Multivariate modeling enabled quantitative prediction of hardness, chewiness, and gumminess with high accuracy (Table 2 and Fig. 7). To our knowledge, this is the first application of three surface hyperspectral data types for TPA prediction in maricultured fish. Moreover, this rapid and non-invasive approach holds promise for application to other economical maricultured fish species. However, due to the interspecific variations in body coloration, scale morphology, skin tissue structure, and muscle biochemical properties, direct model transfer across species would require species-specific calibration or retraining to ensure predictive accuracy and robustness. Importantly, the flexibility and portability of HSI allow for lifetime monitoring and large-scale application, accelerating the selection of superior germplasm with excellent flesh texture.

### 4.3 Advantages of ML modeling analysis based on full spectra for HSI

Three surface hyperspectral data types (400–1000 nm) were used for TPA prediction. While absolute reflectance values differed among SWS, SOS, and RSOS dataset, their spectral characteristics remained consistent, and aligned with previous studies on common carp (Cao et al., 2023; 2024); crispy tilapia (Tang et al., 2024) and grass carp (Ma et al., 2017). Spectral features correspond to molecular overtone and combination vibrations (Zuo et al., 2024). Specifically; the absorption peak around 410 nm is attributed to Soret absorption; whereas peaks at 490; 540 and 580 nm are linked to the pigment absorption (Tang et al., 2024; Xi et al., 2025). The absorption at approximately 970 nm may be related to the O—H stretching vibration (Ecarnot et al., 2013).

Compared with SWS and SOS, RSOS yielded superior performance, we propose that the key difference stems from how each surface preparation modulates light interaction with the underlying tissue. The consistently lower and less variable reflectance of the SWS spectra (as shown in Fig. 3) strongly suggests that the intact scales and pigment act as a dominant, highly scattering layer that obscures detailed spectral information from deeper tissue structures. While scale removal (SOS) eliminates this primary scattering barrier, the spectral features remain subdued and similar to SWS, which we hypothesize is due to the masking effect of skin pigments in the epidermis. In contrast, the RSOS preparation, which involves gently smoothing the skin by wiping from the tail towards the head direction after scale removal, is hypothesized to provide a more optimal window into the muscle properties because it minimizes the specular reflection and strong scattering from the scale layer. Furthermore, by slightly altering the surface condition, it may reduce the immediate interference from superficial pigments, allowing the detection of spectral variations more closely related to the structural and compositional properties of the muscle. This is supported by the greater spectral variability observed in RSOS data and its noted closer resemblance to the spectral profile of fish muscle itself (Tang et al., 2024). Consequently, the RSOS spectra are likely more directly correlated with the structural and compositional properties of the muscle texture.

Hyperspectral image collection process is usually influenced by multiple factors, and the preprocessing methods effectively highlight the spectral features and reduce data variability (Cozzolino et al., 2023; Dong et al., 2023; He et al., 2023). Compared with the original spectrum, the preprocessed spectral data yielded better prediction accuracy in this study (Supplementary table S2). Derivative method (S-G + 1st, S-G + 2nd) are widely used in spectroscopy preprocessing (Xi et al., 2025; Zhou et al., 2025). In our research, S-G + 2nd was optimal for chewiness, springiness, and cohesiveness, while S-G + 1st processed data was superior for hardness and gumminess prediction (Table 2, Fig. 8). The possible reason is that the derivatives emphasize minor spectral differences by removing additive and multiplicative scattering (Rinnan et al., 2009; Vidal et al., 2016).

Five ML methods were used to full spectra to establish prediction models for the TPA parameters in spotted seabass. RF, a relatively simple algorithm, achieved optimal performance for most parameters, whereas, the more complex CNN was only effective for hardness and resilience (Table 2). Similar findings have been reported, with conventional ML methods sometimes outperforming deep learning in HSI modeling (Tang et al., 2025; Cao et al., 2024); potentially due to CNN's requirement for larger datasets (Sharma et al.; 2023). Optimal models for hardness; chewiness; and gumminess predictions (R^2^_P_ > 0.9; RPD > 3.0; MAPE<10%) enabled satisfactory quantitative analysis (Williams; 2014). Models for cohesiveness and springiness (R^2^_P_ ∼ 0.85; RPD ∼ 2.7) are suitable for qualitative analysis. Prediction for adhesiveness and resilience were less accurate; consistent with previous studies; possibly due to their dependence on muscle architecture; connective tissue distribution; moisture and protein content; as well as limited parameter variation in this study (Cao et al., 2023; Wu et al., 2014; Xiong et al., 2015). Other research suggested that 1000–1800 nm might be more suitable for resilience prediction (Zhou et al., 2020). The visualization is a key advantage for HSI (Wu et al., 2014). Distribution maps derived from optimal models revealed spatial TPA variations imperceptible to the naked eyes; reflecting heterogeneous distribution of muscle fibers; intramuscular connective tissue; and intermuscular fat (Wang et al.; 2023; Zhang et al., 2022). These maps provide straightforward visualization of texture spatial characteristics of spotted seabass.

While optimal models show satisfactory results, room for improvement remains. Firstly, using surface hyperspectral data for TPA modeling is a significant advancement over destructive traditional texture analyzer methods. However, RSOS data, requiring scale removal, yielded better predictions than SWS. Future work should focus on advanced spectral processing and ML to enable robust TPA prediction and visualization using non-invasive SWS data. Furthermore, the spectral features directly correlated with individual TPA parameters require further identification to establish causal relationships between TPA parameters and spectral fingerprints.

## 5 Conclusion

This study demonstrates the feasibility of using surface hyperspectral imaging (HSI) combined with ML for the rapid, non-invasive prediction and visualization of TPA parameters in live spotted seabass. The result showed that RSOS data yielded optimal predictions for hardness, chewiness, gumminess, springiness and cohesiveness, meanwhile, SOS was best for adhesiveness and SWS for resilience. RF and CNN were the most effective ML algorithms to predict the TPA parameters. Optimal models for hardness (RSOS-S-G + 1st-CNN), chewiness (RSOS-S-G + 2nd-RF), and gumminess (RSOS-S-G + 1st-RF) predictions enabled satisfactory quantitative analysis (R^2^_P_ > 0.9, RPD > 3.0, MAPE<10%), while, models for cohesiveness (RSOS-S-G + 2nd-RF) and springiness (RSOS-S-G + 2nd-RF) were suitable for qualitative analysis (R^2^_P_ ∼ 0.85, RPD ∼ 2.7). The poor prediction results for adhesiveness and resilience were insufficient for subsequent analysis and needing further improvement. The generated distribution maps successfully visualized spatial textural heterogeneity. However, the reliance on RSOS data, which requires scale removal, presents a limitation for fully non-invasive application, as models based on intact skin (SWS) showed inferior performance. Furthermore, predictions for adhesiveness and resilience remained unsatisfactory. Future work should focus on advanced spectral processing and ML techniques to enhance model robustness using SWS data, and explore the methodology's applicability across diverse aquaculture conditions to promote practical, in-situ texture assessment.

## CRediT authorship contribution statement

**Shuai Che:** Writing – original draft, Validation, Software, Methodology, Formal analysis, Data curation, Conceptualization. **Ang Li:** Validation, Software, Methodology, Formal analysis, Data curation. **Huan Wang:** Validation, Investigation, Data curation. **Changting An:** Visualization, Data curation. **Xiang Fang:** Supervision, Resources. **Qing Wang:** Supervision, Resources. **Qiyou Tao:** Supervision, Resources. **Yun Li:** Writing – review & editing, Supervision, Resources. **Changlin Liu:** Supervision, Project administration, Funding acquisition. **Shufang Liu:** Writing – review & editing, Supervision, Project administration, Funding acquisition, Conceptualization. **Zhimeng Zhuang:** Writing – review & editing, Conceptualization.

## Declaration of competing interest

The authors declare that they have no known competing financial interests or personal relationships that could have appeared to influence the work reported in this paper.

## Data Availability

Data will be made available on request from the corresponding author.
