# Corrigendum to “Quantitative analysis and visualization of live spotted seabass (*Lateolabrax maculatus*) flesh texture using hyperspectral imaging and machine learning” [Food Chem. X 35 (2026) 103773]

**DOI:** 10.1016/j.fochx.2026.104282

**Published:** 2026-08-11

**Authors:** Shuai Che, Ang Li, Huan Wang, Changting An, Xiang Fang, Qing Wang, Qiyou Tao, Yun Li, Changlin Liu, Shufang Liu, Zhimeng Zhuang

**Affiliations:** a State Key Laboratory of Mariculture Biobreeding and Sustainable Goods, Yellow Sea Fisheries Research Institute, Chinese Academy of Fishery Sciences, Qingdao 266071, China; b Laboratory for Marine Fisheries Science and Food Production Processes, Qingdao Marine Science and Technology Center, Qingdao 266237, China; c Fujian Minwell Industrial Co., Ltd., Ningde 355200, China; d South China Sea Fisheries Research institute, Chinese Academy of Fishery Science, Guangzhou 510300, China; e Ocean University of China, Qingdao 266003, China

The authors regret in the Acknowledgements section of the above-mentioned article (DOI: https://doi.org/10.1016/j.fochx.2026.103773), **the grant number of the National Natural Science Foundation of China was inadvertently printed as 3240298. The correct number should be 32402986**. This typographical error does not affect the scientific data, methodology, results, interpretations, or conclusions of the study. All authors have approved this corrigendum.

The authors would like to apologise for any inconvenience caused.

